# Genetic Susceptibility to Tuberculosis and the Utility of Polygenic Scores in Population Stratification

**DOI:** 10.3390/ijms26199544

**Published:** 2025-09-30

**Authors:** Mariia A. Dashian, German A. Shipulin, Andrei A. Deviatkin

**Affiliations:** 1 Federal State Budgetary Institution ‘Centre for Strategic Planning and Management of Biomedical Health Risks’ of the Federal Medical and Biological Agency, 119121 Moscow, Russia; ria.dashyan@gmail.com (M.A.D.);; 2 Department of Biomedicine, Pirogov Medical University, 117997 Moscow, Russia; 3 Laboratory of Postgenomic Technologies, Izmerov Research Institute of Occupational Health, 105275 Moscow, Russia

**Keywords:** tuberculosis, PRS, polygenic score, infection, susceptibility

## Abstract

Tuberculosis (TB) is one of the leading infectious causes of mortality worldwide. Although a significant proportion of the population (up to 36%, depending on the region) is infected with the latent form of TB, only about one in ten of these people will develop an active form of the disease in their lifetime. This is due to a complex interaction between the host’s genetic predisposition and environment. However, the genetic determinants of TB are not well established and have been insufficiently explored in previous genome-wide association studies (GWAS) with sparse and incongruent results. We reviewed recent evidence on host genetic susceptibility to TB, highlighting population-specific characteristics, host–pathogen coevolution, and the limitations of conventional GWAS approaches in terms of clinical and genetic heterogeneity. While rare variants with high penetrance, such as *TYK2* P1104A, lead to monogenic susceptibility, most heritable risk results from the cumulative effect of numerous common variants. This cumulative effect may be summarized using polygenic risk scores (PRSs). Although their use has been proven for non-communicable diseases, PRSs are not applied to infectious disease susceptibility. To date, no PRS model for susceptibility to tuberculosis has been consistently validated. The development of PRSs for TB susceptibility is limited by phenotypic heterogeneity, population structure, and co-adaptation between host and pathogen. Another major challenge is to take into account the considerable influence of environmental factors. This difficulty in modeling environmental influences probably explains the current lack of a clinically applicable PRS for TB susceptibility. However, taking these caveats into account, polygenic models could improve risk stratification at the individual level compared to single-variant association and allow for earlier targeted treatment and prophylaxis.

## 1. Introduction

Some people have severe symptoms of infectious diseases, while others are asymptomatic or recover quickly. It is noteworthy that infected asymptomatic individuals usually do not require medical treatment. As a result, these cases usually remain unnoticed. Therefore, the actual incidence of asymptomatic infections is unknown. For example, it is estimated that approximately 25% of COVID-19 cases worldwide were asymptomatic [1]. Furthermore, studies have shown that unvaccinated and previously uninfected individuals can carry antibodies to dangerous infections such as tick-borne encephalitis [2,3,4] and rabies [5,6]. These cases are rarely studied, and current immunological knowledge largely excludes information about asymptomatic cases. This means that the current understanding of the actual epidemiology of infectious diseases is rather fragmented, as it is unclear what causes differences in disease outcomes.

Tuberculosis (TB) is one of the most socially significant diseases, caused by *Mycobacterium tuberculosis* (MTB) [6]. It is the leading cause of death from an infectious agent [7]. According to estimates, about one-third of the world’s population is infected with MTB; however, only around 5 to 10 percent of these individuals will develop active TB [8]. Importantly, the proportion of individuals with latent infection varies significantly across regions. Rough estimates suggest that 14% of people in North and South America carry latent infections, compared to 36% in Southeast Asia [9]. This disparity likely reflects region-specific combinations of environmental exposures and genetic backgrounds. However, the relative weight of these factors remains unclear. The reasons for variations in susceptibility to infections, including TB, remain poorly understood. Both environmental [10] and genetic factors [11] have an impact on the disease development. Environmental factors such as smoking, poor nutrition, stressful situations, and air pollution can weaken the immune system by impairing lymphocyte function and antibody production [12,13,14,15,16]. At the same time, each person has a unique set of genes that determines individual and population differences in the functioning of immune systems and susceptibility to infections [17]. In Lübeck, Germany, during the 1930 bacille Calmette–Guérin (BCG) vaccination program, 251 newborn infants were mistakenly given the vaccine containing both the attenuated BCG strain and live MTB. Of these, 228 developed some clinical or radiographic evidence of TB disease. Of these children, 72 died, while 156 demonstrated positive tuberculin skin test results but no clinical manifestations of TB [18]. Although the exact MTB dose likely varied among the children, it is reasonable to assume that genetics played a larger role than environmental factors in the manifestation of the disease, given that the children lived in similar conditions and received treatment from the same doctors.

Twin studies are the gold standard for assessing the genetic influence on any trait since monozygotic twins have identical genomes. If the hereditary component of the phenotype is significant, the concordance rate for a disease is higher in monozygotic twins than in dizygotic twins. Twin studies have shown that genetics undoubtedly plays a role in susceptibility to TB. In 1936, the heritability of the incidence of TB was investigated by observing 205 twin pairs, and higher concordance for TB was found in monozygotic (65%) rather than in dizygotic twin pairs (25%) [19]. Similar findings were reported by Comstock et al. [20], in which a multiple regression analysis was conducted in order to eliminate differences caused by factors other than zygosity. Noteworthy, younger twins show lower concordance rates, indicating a stronger environmental role early in life [19,21].

Both genetics and environmental factors [22,23] contribute to TB development, but their relative influence varies among individuals. Schurz et al. [24] indicated a substantial genetic influence on TB risk, with a pooled polygenic heritability estimate of 26.3% across different ancestries. In the presence of genetic predisposition, even minimal environmental exposure may be sufficient to trigger disease onset, whereas individuals with low genetic susceptibility may remain unaffected despite substantial environmental exposure.

Genetic susceptibility to TB has been extensively studied. Previous research has focused on the immunology of MTB infection [8], the impact of rare monogenic variants (e.g., TUK2), and summarizing the results of GWAS performed [24]. However, the applicability of PRS to susceptibility to TB or the challenges that establishing a PRS for susceptibility to TB might pose were not discussed. Therefore, the aim of this review is to summarize recent genetic findings to provide a realistic assessment of the possibilities and limitations of PRS for TB susceptibility.

## 2. Host–Pathogen Coevolution

The genomics of the infection process can be viewed from two perspectives: the pathogen and the host. Different MTB lineages exhibit a high degree of genetic similarity. Moreover, even closely related species within the *Mycobacterium tuberculosis* complex (MTBC)—such as *M. africanum*, *M. bovis*, and others—are genetically nearly indistinguishable (pairwise digital DNA–DNA hybridization—91.2–99.2%; Average Nucleotide Identity: 99.21–99.92%). As a result, it has been proposed to reclassify all members of the MTBC as variants of *M. tuberculosis* [25] (“https://lpsn.dsmz.de/species/mycobacterium-tuberculosis” assessed on 24 September 2025).

Despite such genetic similarity, different Mycobacterium lineages vary in infectivity across ethnic groups [26,27,28,29,30], likely reflecting long-term host–pathogen coevolution. For example, the restricted geographic range of *M. africanum* has been attributed to its coadaptation with specific West African subpopulations [31]. Over evolutionary time, human populations have accumulated unique immune-related genetic variants. Concurrently, certain MTB strains may have adapted to the immune profiles of their local human hosts, shaping population-specific susceptibility to TB.

Experimental evidence for host–pathogen coadaptation was demonstrated using a mouse model of TB susceptibility. The authors infected mice from various inbred lineages with a library of MTB mutants and quantified the relative abundance of each mutant in the input library versus the bacterial population recovered from the spleens one month after infection [32]. Lineage-specific sets of MTB genes required for successful infection were identified for each mouse genotype. These results suggest that different genetic backgrounds of the host exert different selection pressures on the pathogen, supporting the hypothesis of host–pathogen coadaptation. Such mechanisms could explain the regional dominance of certain MTB lineages that may have adapted to the genetic profiles of local human populations. In addition, the population-specific effects of certain human genetic variants on TB susceptibility may be shaped in part by long-term exposure to regionally predominant MTB strains.

## 3. Limited Success of Genome-Wide Association Studies (GWAS) in TB

Totally, 18 genome-wide association studies (GWAS) have been performed to determine the genetic variants responsible for TB risk (Table 1). A systematic PubMed search with the query “Tuberculosis”[MeSH] AND GWAS [MeSH Terms] AND human[MeSH Terms] NOT “Review”[Publication Type] (accessed on 23 May 2025) yielded 116 publications, 17 of which were found to be eligible after manual screening. One additional relevant study [33] was manually included in this meta-analysis, bringing the total number of GWAS on TB susceptibility to 18. The design and sample size of the studies varied considerably, with the number of cases ranging from 197 to 13,692 and the number of healthy controls ranging from 91 to 283,250 participants. These studies covered a variety of populations, including individuals of African (Ghanaian, Gambian, South African), Asian (Han Chinese, Thai, Japanese, Korean), European (Icelandic, Russian), and admixed (Peruvian) origin.

**Table 1:** Overview of GWAS investigating genetic susceptibility to TB.

Reference	Sample Size (Cases/Healthy Controls)	Significant SNPs	Population Ancestry	Year
[34]		rs2243639 (SFTPD)		2014
		rs2569190 (CD14)	South African Colored (SAC)	
	918/507	rs34069356 (CTSZ)		
[35]	410/405	not statistically significant	South African Colored (SAC)	2019
[36]	642/91	rs2057178	South African Colored (SAC) population	2014
[37]	5530/5607	rs4733781 (ASAP1) rs10956514 (ASAP1) No associations were observed for rs10956514 and rs11774633 (ASAP1) with TB in Chinese populations [38]	Russian (replication in the African population)	2015
[39]	Four datasets: young Japanese (60/249), young Thai (137/295), old Japanese (123/685), old Thai (300/295)	rs6071980	Thai/Japanese	2012
[40]	4310/6386	rs41553512 (HLA-DRB5)	Han Chinese	2017
[41]	2237/3122	rs4331426 (LOC124904262: Intron Variant) No associations were observed for rs4331426 with TB in Chinese populations [38]	Ghanaian/Gambian	2010
[33]	8821/13,859	rs2057178	Ghanaian/Gambian (Russian/Indonesian)	2012
[42]	333/616	rs17155120 (C10orf90: Intron Variant)	Vietnamese/French/South African	2021
[43]	13,692/283,250	rs557011	Icelandic/Russian/Croatian	2016
[44]	646/1813	rs9365798	Korean	2017
[45]	616/709	rs3751112 (RAB17), rs141645096 (DCTN4)	Han Chinese	2021
[46]	2175/1827	rs73226617 (LINC02618: Intron Variant)	Peruvian (Lima)	2019
[47]	2949/5090	rs12437118 (ESRRB), rs6114027 (TGM6: Intron Variant)	Han Chinese	2018
[48]	556/650	rs916943 (AGMO: Intron Variant)	Moroccan/Russian	2016
[49]	2653/2537	rs2273051 (COL4A5)	Indonesian/Russian	2012
[50]	4426/84,290	rs2894257	European	2017
[51]	43 patients with active TB and 49 with latent TB/313 controls	rs62292160	Chinese Han	2021

Key findings include loci in HLA genes, macrophage regulators (ASAP1, MAFB), and monocyte-specific enhancers (ATP1B3). However, only a few SNPs were replicated across different studies (Table 1). The WT1 locus, identified in cohorts from Ghana and Gambia, was replicated in cohorts from South Africa and Russia. The ASAP1 locus, first reported in a Russian cohort, was later validated through a reanalysis of previous datasets [27,52]. The human leukocyte antigen (HLA) region, particularly the HLA class II subregion, has been identified as a significant correlate of TB susceptibility [24,53].

It should be noted that GWAS findings often contradicted each other and failed to be replicated in independent studies (Table 1). There are several possible explanations for this observation.

Firstly, TB is a complex polygenic disease influenced by many genetic variants, each exerting a small effect. Detecting these weak signals is challenging with conventional GWAS methods. Genetic susceptibility to TB results from mutations that impair the function of numerous genes that are important for immune protection. Significant genetic changes not only take place in regions of genes that code for proteins but also in non-coding regions. These regions and regulatory protein-coding genes, such as transcription factors, play a significant role in immune function [54,55]. In theory, different groups of people exposed to TB can carry different mutations in different genes—for example, one group may carry mutations in interferon genes, another may carry mutations in HLA variants, and a third group may carry mutations in transcription factors regulating other immune genes. Once different groups of people with different genetic mutations are mixed, the signals from single variants overlap, and it becomes more challenging for GWAS to detect them.

Furthermore, TB presentation differs among individuals, diverging throughout investigations. Active TB includes various clinical forms, potentially exhibiting distinct genetic origins [56]. Although pulmonary TB persists as the predominant manner of its manifestation, the infection may essentially involve each organ system. Even within pulmonary cases, the disease progression differs, with some individuals developing symptoms shortly after primary infection, whereas others experience a reactivation of latent TB years later. This clinical and biological diversity complicates the definition of a consistent phenotype for genetic analysis [57] and may account for the poor replicability observed in TB GWAS.

GWAS have been performed across populations with diverse ethnic backgrounds (Table 1), which differ in allele frequencies and patterns of linkage disequilibrium. Consequently, genetic associations identified in one population are frequently not applicable in others. Different populations may have distinct components of the immune response affected, resulting in varied associated genetic variants [53,58]. For example, the IL-10 promoter polymorphism rs1800896 increases TB susceptibility in Caucasian but not in African populations [59]. Moderate inflammation generally protects against the development of TB, while chronically increased inflammation is strongly associated with disease progression, regardless of bacterial load [60]. Ethnic differences in inflammatory profiles have been reported, with individuals of African descent generally exhibiting higher systemic inflammation than those of European ancestry [61]. This tentatively suggests that higher anti-inflammatory cytokine levels may suppress inflammatory responses in individuals of European origin, thereby increasing their susceptibility by impairing host defense. Conversely, elevated pro-inflammatory cytokine levels in individuals of African ancestry may exacerbate inflammation, increasing TB risk in genetically predisposed persons.

Population heterogeneity and limited sample sizes likely contribute to inconsistent GWAS findings. However, even larger studies combining multiple GWAS datasets reported ethnic disparities. There was only one SNP that reached genome-wide significance for the mixed ancestry dataset, but once the dataset was split into different populations, more prospective SNPs were found for the European and Asian populations separately [24]. Ancestral migrations, infectious disease selective pressures, genetic drift, and local adaptations have led to unique genetic susceptibility patterns among ethnic groups for TB. Thus, populations harbor distinct alleles modulating immune responses to MTB. Moreover, identical SNPs may be functionally distinct in individuals with different ancestry [59]. Such variability further complicates the replication of SNP–disease associations across ethnically diverse studies. Environmental factors additionally modulate the expression of genetic susceptibility. Factors such as nutrition, air quality, co-infections, and access to healthcare can mask or amplify the effect of genetic variants [44]. These variables add complexity to genetic studies of TB. The most plausible explanation for the poor reproducibility of GWAS results in TB susceptibility is therefore the high heterogeneity of the disease. Broader approaches, such as polygenic risk scores (PRS), may offer a more accurate and robust way to assess inherited susceptibility to TB across various populations.

## 4. Genetic Susceptibility to TB Is Caused by a Continuum Ranging from Monogenic to Polygenic Risk

Unlike severe congenital immunodeficiencies, TB in developed countries with access to effective treatment exerts only modest selective pressure. Even untreated, this disease typically progresses slowly over several years, gradually impairing quality of life rather than causing rapid mortality. Although maternal morbidity in individuals with active TB is increased 2.8-fold and perinatal mortality is 4.2 times higher, the majority of offspring survive, allowing for deleterious alleles to persist in the population without being effectively purged by selection [62].

However, in contexts of intense transmission and high mortality, strong negative selection may occur. For example, after TB was introduced to a Native American reservation in Saskatchewan in 1890, the first-year mortality rate reached 10% of the population. Over the subsequent three generations, more than half of the initial families perished. However, by 40 years after the epidemic began, annual mortality had dropped to 0.2%, consistent with a possible selective sweep that presumably removed highly susceptible individuals [63].

Genetic evidence of such selection was demonstrated for the *TYK2* P1104A variant (rs34536443), identified as a monogenic risk factor for TB [64]. *TYK2* encodes a non-receptor tyrosine kinase of the Janus kinase family, mediating signal transduction downstream of cytokines including IL-12, IL-23, IFN I, IL-6, IL-10, and IL-13. *TYK2*-deficient T cells display impaired responses to IL-12, IL-23, IFN-α, and IL-10, resulting in increased susceptibility to MTB and viral infections [65]. At the same time, the rs34536443 polymorphism is associated with protection against multiple autoimmune diseases, such as systemic lupus erythematosus and multiple sclerosis. However, individuals homozygous for P1104A are at a significantly increased risk for clinical TB progression due to IL-23-dependent antimycobacterial IFN-g immunity, which these individuals lack. Homozygosity for P1104A is the first common monogenic cause of TB that has been identified. The current frequency of the P1104A allele in European populations has significantly decreased compared to ancient European DNA samples. These findings suggest that negative selection against the *TYK2* P1104A allele by endemic TB in Europe may have contributed to a slow genetic purge of this susceptibility allele during recent millennia [55,64].

Mendelian susceptibility to mycobacterial disease (MSMD) is a rare genetic disorder characterized by impaired immunity against intracellular pathogens, such as MTB, attenuated vaccine strains, and environmental mycobacteria in otherwise healthy individuals. Genetic variants in 19 genes (*IFNG*, *IFNGR1*, *IFNGR2*, *STAT1*, *IL12B*, *IL12RB1*, *IL12RB2*, *IL23R*, *RORC*, *TBX21*, *IRF8*, *SPPL2A*, *ISG15*, *USP18*, *TYK2*, *JAK1*, *ZNFX1*, *NEMO*, and *CYBB*) lead to MSMD and define 34 disorders that reflect high levels of allelic heterogeneity. The pathogenesis of MSMD depends mostly on the gene variant that leads to either insufficient production or inadequate response to interferon gamma (IFN-γ), which is an indispensable part of an efficient immune response against mycobacteria [66,67]. Biallelic variants in *IL12RB1* are the most frequent genetic cause, being present in approximately 60% of patients diagnosed with MSMD [68].

## 5. The Potential and Challenges of PRS for TB

**Figure 1:**
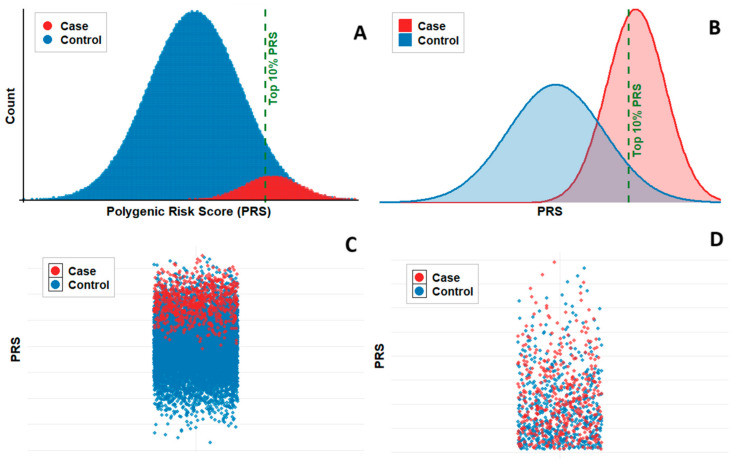
Visualization of possible PRS distribution among cases and healthy controls. (**A**) Dot plot of individual PRS values for cases (red) and healthy controls (blue). Each point represents one individual; points are jittered vertically for clarity. The dashed green vertical line indicates the 90th percentile threshold (top 10% PRS). (**B**) Kernel density estimates of PRS distributions for cases and controls. The distribution for cases is shifted rightward, indicating higher genetic risk scores in this group. The dashed green line marks the top 10% PRS cutoff. (**C**) Jitter plot of PRS values for all individuals grouped by status, showing the spread and overlap of scores between cases and controls. (**D**) Jitter plot of PRS values restricted to individuals within the top 10% PRS percentile. This highlights the enrichment of cases among high-risk individuals. Although this is a generic example, it illustrates why PRSs are relevant for tuberculosis: while no single variant confers a strong effect, the cumulative distribution of many variants can stratify individuals by their probability of progressing from latent infection to active TB.

TB infection is a phenotypic marker of susceptibility that manifests when environmental and infectious exposures align with permissive host factors. The genetic causes can vary across populations and individuals due to immune system complexity, genetic heterogeneity, and environmental variability. Despite efforts, GWAS have not identified reproducible loci for TB sensitivity. These inconsistencies highlight the complex genetic architecture of TB sensitivity, whereby numerous common variants with small effects collectively influence susceptibility. More integrative approaches, such as PRS, aim to estimate genetic predisposition to a specific trait by aggregating the effects of numerous common genetic variants [69]. This metric is calculated as the weighted sum of risk alleles, with each allele representing an estimated contribution to disease risk. It is important to note that current PRS models for complex traits are primarily derived from populations of European ancestry. Their transferability to other populations, especially those most affected by TB, is a major unsolved challenge [70]. More sophisticated models of PRS estimation incorporate linkage disequilibrium to account for correlations between SNPs. It should be noted that unlike monogenic mutations with high penetrance, a high PRS is not a precise criterion for the presence of the selected trait. Rather, as illustrated in Figure 1, PRS provides a probabilistic stratification that may be particularly informative for tuberculosis, where the cumulative burden of many common variants rather than a single strong-effect allele underlies disease susceptibility. Indeed, PRSs facilitate genetic risk stratification by quantifying the cumulative burden of risk alleles.

## 6. Limited Application of PRSs to Infectious Diseases

PRSs are widely used to stratify genetic risk for non-communicable diseases—cardiovascular and metabolic disorders, multiple types of cancer, and autoimmune conditions such as rheumatoid arthritis and type 1 diabetes. In some cases, individuals in the top decile of PRS distribution exhibit risks comparable to those seen in monogenic disorders [71]. It should be noted that attempts are already being made to introduce PGS for ten diseases into routine clinical practice [72]. Importantly, PRS-based stratification enables targeted preventive interventions. For example, the onset of type 1 diabetes (T1D) may be prevented by immunomodulatory treatment such as teplizumab, a monoclonal anti-CD3 antibody, when administered at the preclinical stage identified by the presence of autoantibodies [73]. However, population-wide autoantibody screening is cost-inefficient. By contrast, pre-screening based on PRS enables the selection of a genetically high-risk subgroup for further immunological testing [74]. Notably, the T1D PRS model demonstrated that the top 10% of genetically at-risk individuals accounted for over 77% of all T1D cases [75].

Despite progress in other disease areas, PRSs remain largely unused in the context of infectious diseases. The PGS Catalog (https://www.pgscatalog.org/ (accessed on 1 August 2025)), a curated repository of published PRS models, shows a highly uneven distribution across disease classes (see Table 1). As of May 2025, infectious diseases were grossly underrepresented compared to, for example, cancer, cardiovascular disease, and digestive system disease.

This underrepresentation is likely attributable to several factors: the historical focus on monogenic germline errors of immunity, the complexity of incorporating pathogen exposure into genetic models, and the assumption that infections are primarily environmental rather than polygenic traits. However, TB demonstrates consistent evidence of heritable susceptibility and variability in immune response, as do other infections. TB does not follow a purely stochastic course but, rather, emerges at the intersection of exposure and host permissiveness. This provides a strong rationale for applying PRS-based modelling to TB. The lack of reproducible associations in GWAS should not be interpreted as a methodological failure but, rather, as an indicator of the underlying polygenic architecture. Many variants may have a small effect; each one is insufficient on its own, but they contribute to susceptibility when considered together. In such settings, PRSs offer a more realistic framework than attempts to identify “the TB susceptibility” gene. The field of infectious disease genetics may benefit from shifting toward cumulative models that do not rely on strong-effect variants alone.

Despite the well-documented genetic component of infectious disease susceptibility, no PRS for TB had been registered in the PGS Catalog as of May 2025. Meanwhile, hundreds of PRS models for non-communicable conditions have been developed and curated. This underrepresentation is also evident in the scientific literature. A PubMed query “(tuberculosis) AND (polygenic risk score)” returned twenty-nine articles, of which only one [76] was relevant. A broader search that included all infectious diseases was used with the following query: “(“communicable diseases”[MeSH Terms] OR (“communicable”[All Fields] AND “diseases”[All Fields]) OR “communicable diseases”[All Fields] OR (“infectious”[All Fields] AND “disease”[All Fields]) OR “infectious disease”[All Fields]) AND ((“polygenic”[All Fields] AND “risk”[All Fields] AND “score”[All Fields]) OR “polygenic risk score”[All Fields])”. This search yielded ninety-five papers, of which only four [77,78,79,80] are relevant to the application of PRS in infectious disease settings. This reflects both a conceptual gap and a missed opportunity to extend risk stratification tools into the domain of communicable diseases.

Despite the well-documented genetic component of infectious disease susceptibility, no PRS for TB had been registered in the PGS Catalog as of May 2025, which explains the ‘0’ entry in Table 2. At the same time, a few studies [43,72] have tested polygenic models for TB prediction, but these remain proof-of-concept and are not included in curated resources such as the PGS Catalog. One study showed that combining ten common genetic variants with basic clinical information, such as age, sex, and BMI, noticeably improved the ability to predict pulmonary TB development [44]. Another study revealed that variants in TLR1, TLR2, and MMP8 genes helped distinguish between acute and chronic forms of TB. Using these variants, the authors created a PRS that successfully classified individuals into the two clinical groups [76].

## 7. Shared Immunogenetic Components in PRS Across Infectious Diseases

Although PRSs are often specific to a particular trait, susceptibility to infectious diseases may share common genetic components, especially in genes involved in immune signaling. Such susceptibility may arise from impairments in genes encoding proteins with broad defensive functions. For example, one variant may disrupt epitope recognition, and another may interfere with innate immune signaling, both increasing vulnerability to multiple pathogens that rely on these mechanisms. At the same time, infections differ in tissue specificity and pathogenesis. Intestinal infections, for example, may be more frequent in subjects with genetically weakened local immunity of the gut, while respiratory infections may preferentially attack patients with weakened pulmonary defense. Therefore, the character “susceptibility to infection” should include both loci shared by many diseases and loci specific to certain pathogens.

A striking example comes from a study of the TLR1/6/10 locus, where the TLR10 N241H (rs11096957) polymorphism was associated with reduced monocyte activation compared to individuals without this SNP [81]. This highlights how variation in immune-related genes can alter the cellular response to infection and potentially shift disease outcomes. The authors developed an algorithm capable of inferring immune cell activation states from bulk RNA-seq data of patients infected with *Salmonella*. They then applied this model to longitudinal transcriptomic data from TB patients (Berry London: GSE107991; Berry South Africa: GSE107992; Leicester: GSE107994). Remarkably, the monocyte activation profile distinguished latent individuals who remained healthy from those who progressed to active TB already at baseline, before any clinical symptoms emerged. This illustrates how genetically programmed immune responses can influence infection trajectory across different diseases, supporting the notion that some components of PRS for infectious diseases may reflect shared underlying immunogenetic architecture.

## 8. Methodological Challenges in Constructing PRS for Infectious Diseases

The construction of PRS for infectious diseases requires a methodological rethinking, as the direct transfer of approaches successfully applied to non-infectious and chronic conditions will most likely be insufficient. This is due both to the biological nature of infection and to the architecture of interactions between host genes, the pathogen, and the environment.

First, infectious diseases rarely represent a single phenotype. For example, tuberculosis can be asymptomatic (latent), manifest as an active or disseminated form, have a chronic course, or cause rapid progression. Different forms of the disease may be associated with different sets of genetic factors. Therefore, when forming cohorts, it is important to account for clinical heterogeneity—combining all “TB” cases without specifying the forms leads to signal dilution and reduced association power.

Second, ethnic and population structure play a special role. Unlike oncology or diabetes, infectious diseases develop in the context of active interaction with a pathogen. There is compelling evidence of the co-evolution of MTB lineages and human populations. The same gene variant may have different effects on risk depending on the pathogen lineage it interacts with; analyzing without accounting for population stratification and the pathogen’s genetic background is methodologically vulnerable. Therefore, in the early stages of PRS analysis, it is preferable to use phenotypically and genetically homogeneous subsamples.

Third, predisposition to infectious diseases often has a pronounced polygenic nature: the contribution of an individual variant is small, but the cumulative effect across multiple SNPs can be significant. At the same time, different individuals may be susceptible for different reasons: some due to impaired innate immunity (e.g., signaling through TLR1/TLR2), and others due to adaptive immunity, or, say, features of inflammatory regulation. This requires careful SNP selection and a preference for methods that account for linkage disequilibrium (LD), such as PRS-CS [70], LDpred [82], lassosum [83], and other LD-aware approaches.

The fourth important aspect is the combination of genetic and non-genetic factors. Models that use only genetics may lack predictive power. Adding age, sex, HIV status, body mass index, socioeconomic status, and environmental variables can improve the results. This is especially important for infectious diseases whose prevalence is predetermined by external factors to a significant extent.

Finally, the effectiveness of PRS is proportional to the size and diversity of the training set. For most infectious diseases, including TB, such large cohorts with high-quality genetic and phenotypic data are extremely scarce today. The lack of multi-ethnic data is particularly acute, without which it is impossible to build universal models suitable for application outside a specific population. In this context, international consortia and multicenter initiatives play a particularly important role.

## 9. Conclusions

TB remains one of the most common infectious pathogens in the world despite the availability of effective treatment. Meanwhile, it is known that not all patients infected with MTB develop the active form of the disease: in the majority of cases, the disease is latent. Identification of people with increased genetic predisposition towards developing from latent to active infection would most helpfully impact early prevention and target medical attention; e.g., preventive treatment or intensified surveillance. TB is a classic multifactorial disease, where the contribution of individual genetic variants can be relatively small but the cumulative effect of multiple polymorphisms can be significant. This makes it a suitable strategy to use PRS as a risk prediction tool. If the aforementioned challenges can be overcome, a reliable PRS might substantially improve the effectiveness of screening programs, especially in endemic regions.

The development of PRS for TB has been previously hindered for various reasons. First, there was a lack of large, well-defined, and ethnically diverse cohorts available for genetic studies: TB occurs most frequently in resource-poor settings, where the infrastructure for large-scale genetic research is underdeveloped. Second, due to high ethnic heterogeneity, the genetic architecture of risk for TB susceptibility varies significantly between populations, complicating the creation of a universal scoring system. Third, TB exposure is highly dependent on environmental factors—social, economic, and epidemiological status could mask genetic correlations, reducing the predictive power of purely genetic models. Finally, in polygenic risk prediction, priority has long been given to diseases with more apparent genetic contribution to pathogenesis, i.e., cardiovascular diseases, diabetes, or cancer. However, in the new era of the accumulation of large genetic data, the advancement of multi-ethnic research, and the construction of methods for integrating genetic and clinical factors, it has become possible to check the validity of a PRS for TB susceptibility. Such a system could significantly increase the identification of probable future cases of the disease, maximize prevention, and, in the long term, contribute to reducing the global burden of TB, though this remains a speculative goal requiring further validation.

## Figures and Tables

**Table 2:** Number of polygenic scores (PGS) by disease category (PGS Catalog, May 2025).

Cancer	746 PGS
Cardiovascular disease	413 PGS
Digestive system disorder	430 PGS
Immune system disorder	232 PGS
Infectious disease	8 PGS (https://www.pgscatalog.org/trait/EFO_0005741/) (accessed on 1 August 2025)
COVID-19	3 PGS
Tuberculosis	0 PGS
